# Supplementary material for: Archaeal Lipids Regulating the Trimeric Structure Dynamics of Bacteriorhodopsin for Efficient Proton Release and Uptake
Source: Int J Mol Sci. 2022 Jun 21;23(13):6913. doi: 10.3390/ijms23136913 (PMC9278134; doi:10.3390/ijms23136913)
Supplement: Supplementary file 1 [file ijms-23-06913-s001.zip › ijms-1755451-supplementary.pdf]

Supplementary Materials

Archaeal Lipids Regulating the Trimeric Structure Dynamics of Bacteriorhodopsin for Efficient Proton Release and Uptake

Sijin Chen [1,†], Xiaoyan Ding [1,2,†], Chao Sun [1], Fei Wang [1], Xiao He [3,4,*], Anthony Watts [2,*], and Xin Zhao [1,*]

[1] School of Physics and Electronic Science, East China Normal University, 500 Dongchuan Road, Minhang District, Shanghai 200241, China
[2] Department of Biochemistry, University of Oxford, South Park Road, Oxford OX1 3QU, UK
[3] School of Chemistry and Molecular Engineering, East China Normal University, Shanghai 200062, China
[4] New York University-East China Normal University Center for Computational Chemistry, NYU Shanghai, Shanghai 200062, China
* Correspondence: xiaohe@phy.ecnu.edu.cn (X.H.), anthony.watts@bioch.ox.ac.uk (A.W.), and xzhao@phy.ecnu.edu.cn (X.Z.)
† These authors contributed equally to this work.

Materials and Methods

Expression of recombinant bR K40L and K129L mutants

The bR gene fragment termed bop was amplified from the Halorubrum salinarum R1M1. The mutant genes of K40L-bR and K129L-bR were prepared through overlap PCR and named *K40L-bop* and *K129L-bop*, respectively. All of the forward and reverse primers are shown in Table S7. The mutant genes were then combined with the promoter of bop and cloned into the *E. coli* plasmid vector pUC19 to produce the shuttle plasmid *pUC19-bop/K40L/K129L*. The shuttle plasmid was amplified and subcloned into the expression vector to yield the pXLNor-*bop* plasmid by restriction endonucleases *BamH I/Hind III* [1-3]. The expression vector pXLNor-*bop* was transformed into H. salinarum L33 [4] to produce the recombinant proteins, denoted as RC^{L33}-bR, $K40L^{L33}$-bR, and $K129L^{L33}$-bR. The culture and isolation of recombinant proteins were carried out according to the standard protocol, as reported by Oesterhelt et al. and Ming et al. [5, 6]. Briefly, the antibiotic novobiocin was used to screen transfected L33 strains. The 25 mL resistant strain cultures from a single colony were then cultured on a large scale for further expression and purification, and the purple membrane was harvested. A sucrose gradient with concentrations of 35%, 43%, and 60% was used for the purification of the purple and claret membrane. The concentration of the purified protein was determined according to the protocol described previously by measuring the absorbance of A280 of the wild and mutant bR.

Light-induced transient absorption change spectroscopy

The proton-pumping activities of RC^{L33}-bR, $K40L^{L33}$-bR, and $K129L^{L33}$-bR were monitored through light-induced absorption changes using the pH-sensitive dye pyranine (8-hydroxy-1, 3, 6-pyrenetrisulfonic acid, trisodium salt) on a homemade apparatus [6] at 456 nm. The kinetics of the M state, the O state, and the recovery trajectory to the ground state were monitored at 412 nm, 660 nm, and 570 nm, respectively. All experiments were conducted using a photoflash with a half-bandwidth of less than 1 ms for excitation. The time constants were extracted through the best fitting of the experimental data by the multi-exponential functions. All the samples were suspended in a buffer with 100 mM NaCl and 20 mM KCl at pH 7.0 and converted into the light-adapted conformation before being used. All measurements were performed at room temperature.

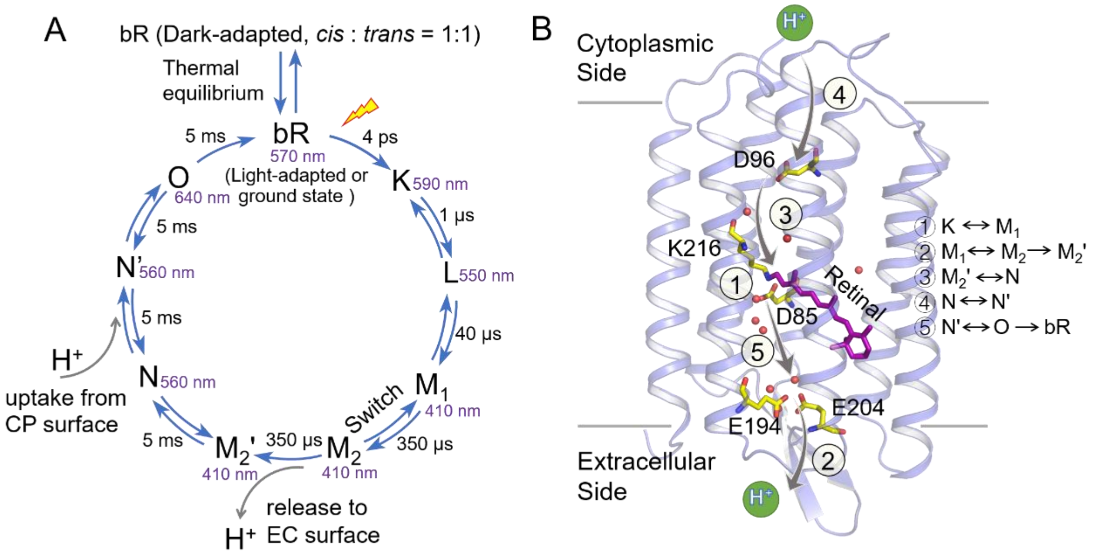

Figure S1. Schematic diagram of bacteriorhodopsin (bR) photocycle and the proton transfer pathway. **(A)** bR photocycle and **(B)** proton transfer pathway. The bR structure of 1C3W (PDB code) is used here. The retinal chromophore possesses a thermal equilibrium between 13-*cis*, 15-*syn* (bR_{cis}), and all-*trans*, 15-*anti* (bR_{trans}) isomers in the dark-adapted bR, whereas the light-adapted protein only contains the all-*trans* isomer. Absorption of a photon by bR initiates a catalytic cycle and leads to the vectorial transport of a proton out of the cell through 5 steps. The process can be described as the retinal first photo-isomerizing from all-*trans* to 13-*cis* configuration (light-adapted to K), followed by a proton transfer from the Schiff base (SB) to the proton acceptor D85 (L to M_1, step 1). Concomitantly, a proton is released to the bulk phase by a group amino acid residue, and the Schiff base changes its accessibility from the extracellular to the intracellular direction (M_1 to M_2, step 2). The Schiff base is reprotonated from D96 in the cytoplasmic channel to allow vectorial transport of a proton (M_2 to N, step 3). After reprotonation of D96 from the cytoplasmic surface (N to O, step 4), the retinal isomerizes thermally, and the accessibility of the Schiff base switches back to extracellular (O to light-adapted, step 5). The extracellular part of bR exhibits a hydrogen-bonded network of charged amino acids and water molecules as potential proton translocation pathway elements. Very few charged amino acids and water molecules are located in the cytoplasmic domain [7-19].

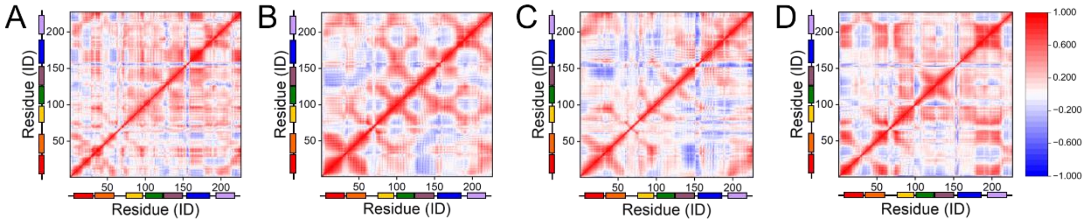

Figure S2. Dynamical cross-correlated map (DCCM) analysis of the Cα atoms from the second-time repeated simulation: **(A)** M1 (Membrane containing the bR trimer, POPC, S-TGA-1, and PGP-Me lipids); **(B)** M2 (Membrane containing bR trimer and POPC lipids); **(C)** M3 (Membrane containing the bR trimer, POPC, and S-TGA-1 lipids); and **(D)** M4 (Membrane containing the bR trimer, POPC, and PGP-Me lipids). The color scale represents the correlation intensity, from the maximum negative (-1) to the ultimate positive (+1).

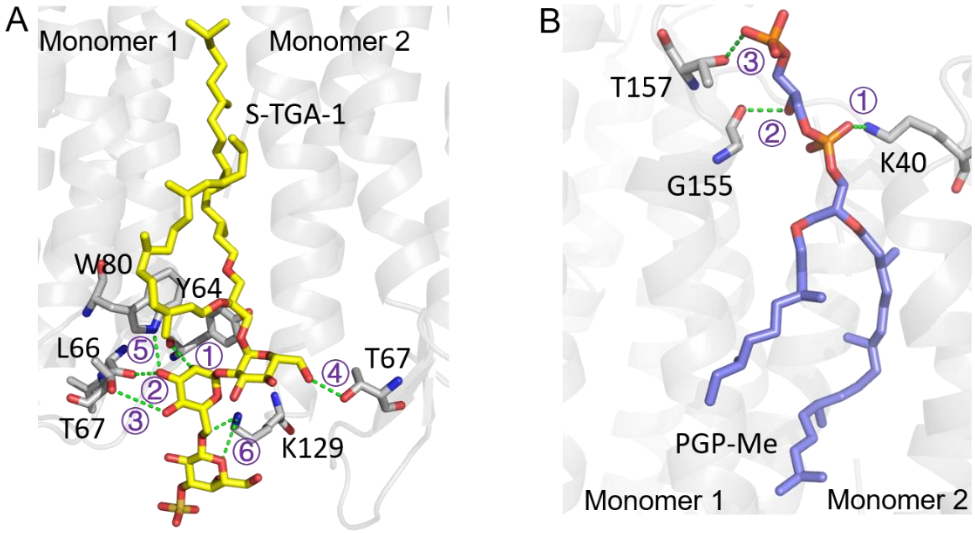

Figure S3. The possible specific H-bond interactions (green dotted line) between S-TGA-1 **(A)** and PGP-Me **(B)** with bR from the last snapshot of the second-time repeated simulations. The grey sticks represent residues that interact with S-TGA-1 or PGP-Me by hydrogen bond, and yellow and dark blue sticks represent S-TGA-1 and PGP-Me.

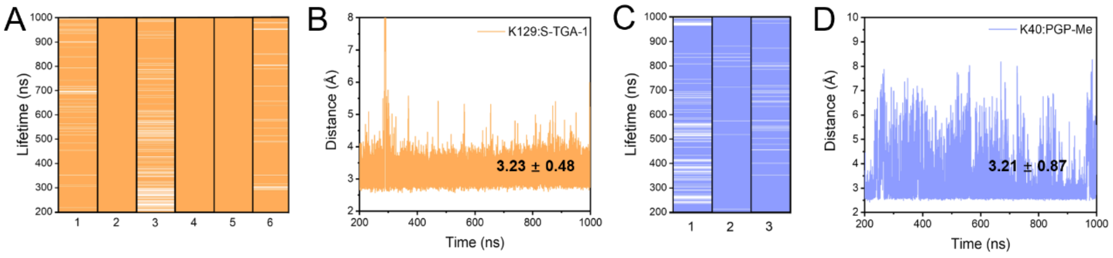

Figure S4. The hydrogen bond and salt bridge evolution throughout the simulations for S-TGA-1 and PGP-Me from the second-time repeated simulations. **(A,C)** Evaluation of the lifetimes of the H-bond interactions for S-TGA-1 **(A)** and PGP-Me **(C)**. **(B,D)** Evolution of distance between K129 and the galactosyl-3-sulfate of S-TGA-1 **(B)**; K40, and the polar headgroups of PGP-Me **(D)** throughout the whole simulation.

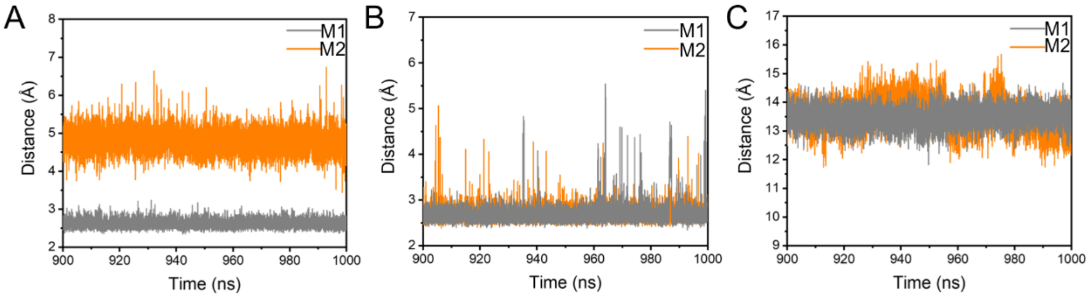

Figure S5. The evolution of distance between the key residues in the last 100 ns of repeated simulations of M1 (grey line) and M2 (orange line): **(A)** E194-E204; **(B)** Y83-E194; and **(C)** R82-A51.

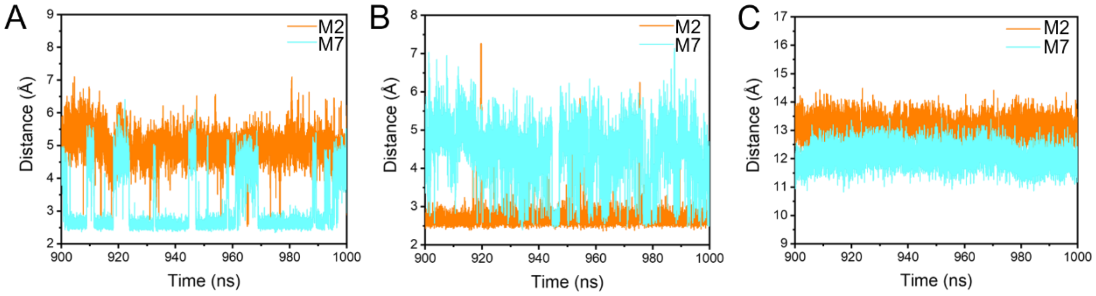

Figure S6. The evolution of distance between the key residues in the last 100 ns of repeated simulations of M2 (orange line) and M7(Membrane containing W80A-bR trimer and POPC lipids, pale cyan line): **(A)** E194-E204; **(B)** Y83-E194; and **(C)** R82-A51.

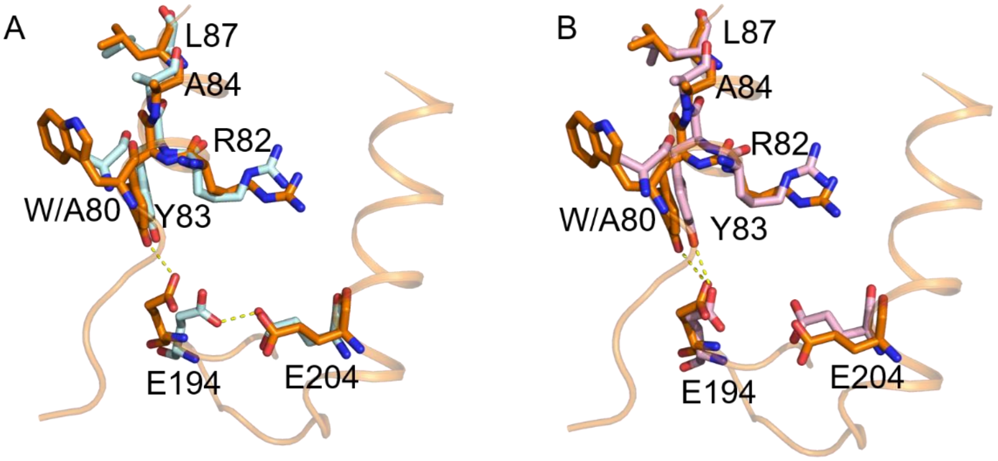

Figure S7. Superimposed drawing of two possible extracellular detailed conformational changes on the extracellular side in M2 (orange cartoon and sticks) and M7 (pale cyan and pink sticks).

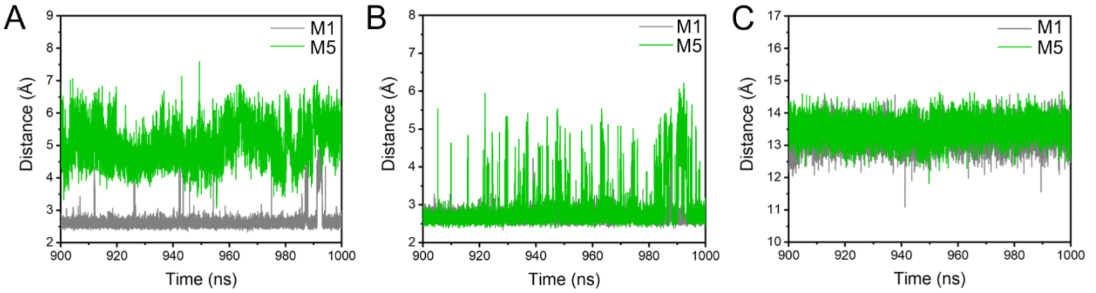

Figure S8. Dynamic changes in key residues by W80A in the second-time repeated simulation. **(A-C)** The evolution of distance between the key residues from the last 100 ns simulations of M1 (grey line) and M5 (Membrane containing the W80A bR trimer, POPC, S-TGA-1, and PGP-Me lipids, green line): **(A)** E194-E204; **(B)** Y83-E194; and **(C)** R82-A51.

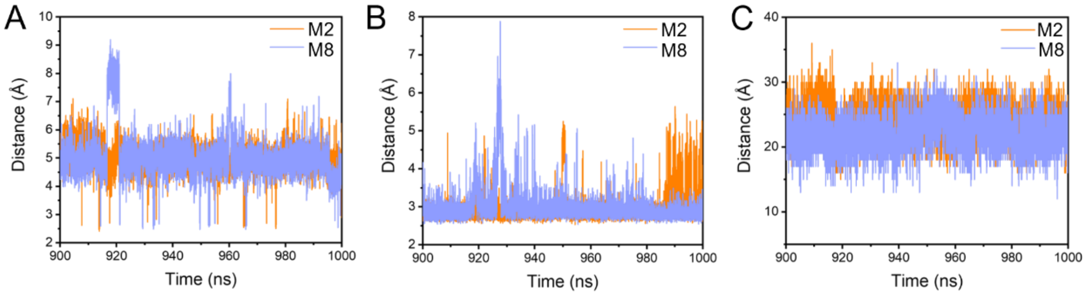

Figure S9. Dynamic changes in key residues and water molecules in M8 (Membrane containing the K129L-bR trimer and POPC lipids only): **(A,B)** The evolution of distance between the key residues from the last 100 ns simulations of M2 (orange line) and M8 (light blue line): **(A)** E194-E204; **(B)** R134-E194. **(C)** Time series of the number of waters around K/L129 and T128 in the last 100 ns simulations of M2 and M8.

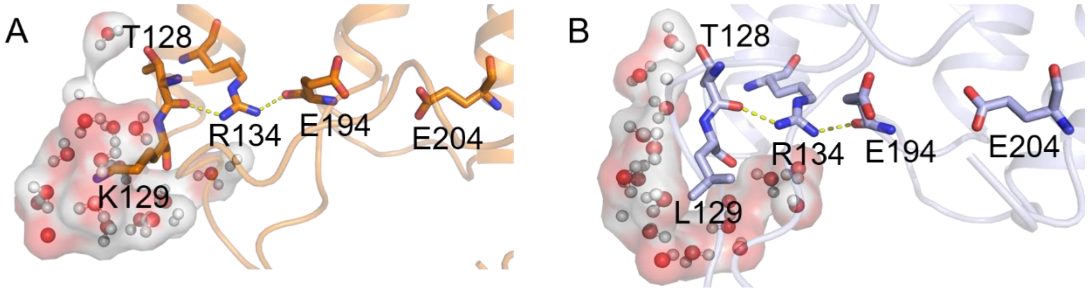

Figure S10. Schematic diagrams of H-bond networks connecting K129 and proton release complex (PRC) in M2 **(A)** and M8 **(B)**.

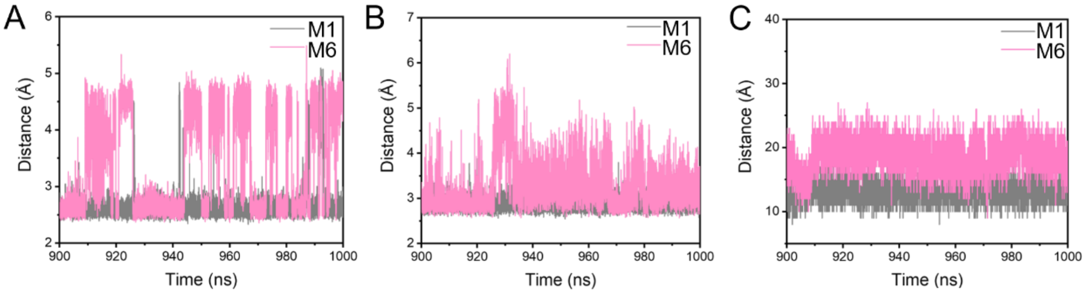

Figure S11. Dynamic changes in the key residues and water molecules by K129L in the second-time repeated simulation. **(A,B)** The evolution of distance between the key residues from the last 100 ns simulations of M1 (grey line) and M6 (Membrane containing the K129L-bR trimer, POPC, S-TGA-1, and PGP-Me lipids, pink line): **(A)** E194-E204; **(B)** R134-E194. **(C)** Time series of the number of waters around K/L129 and T128 in the last 100 ns simulations of M1 (grey line) and M7 (pink line).

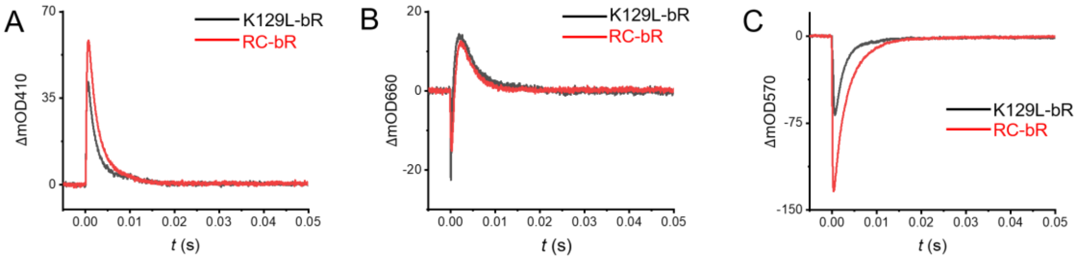

Figure S12. Light-induced transient absorption changes by K129L mutant in the native membrane in the M **(A)** and O state **(B)** and the recovery trajectory towards the bR ground state **(C)** at 410 nm, 660 nm, and 570 nm, respectively.

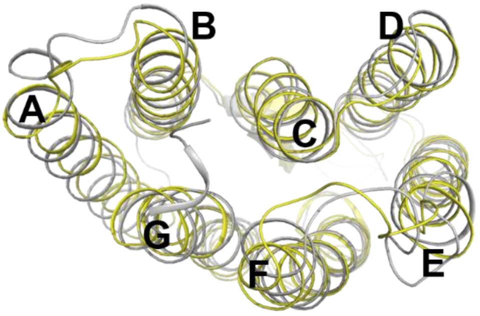

Figure S13. Superimposed drawing of cytoplasmic side of M1 and bR crystal structure containing PGP-Me (PDB ID: 2AT9, yellow cartoon).

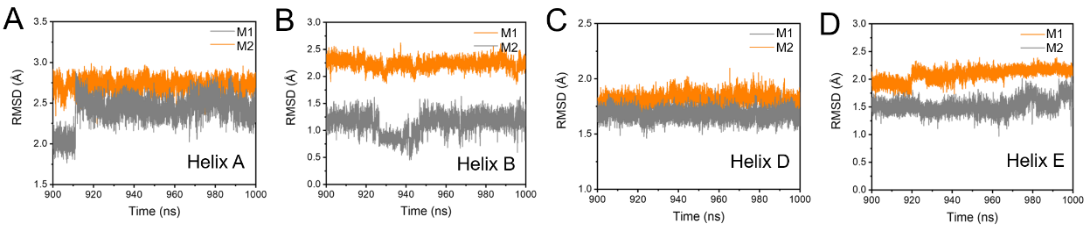

Figure S14. The RMSD for the cytoplasmic ends of helices A **(A)**, B **(B)**, D **(C)**, and E **(D)** during M1 (grey line) and M2 (orange line), regarding a late M-state crystal structure (PDB ID: 1FBB). The smaller RMSDs indicate larger openings.

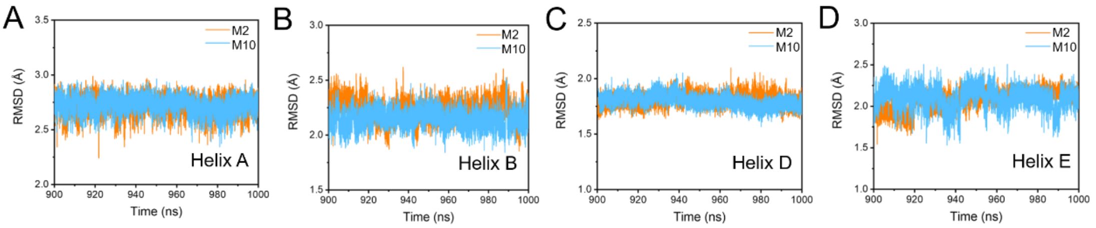

Figure S15. The RMSD for the cytoplasmic ends of helices A **(A)**, B **(B)**, D **(C)**, and E **(D)** during M2 (orange line) and M10 (Membrane containing the K40L-bR trimer and POPC lipids only, sky blue line), regarding a late M-state crystal structure (PDB ID: 1FBB). The smaller RMSDs indicate larger openings.

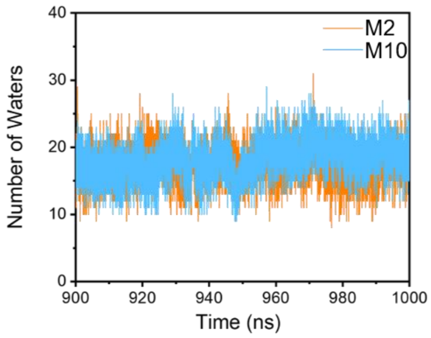

Figure S16. Time series of the number of waters near the cytoplasmic surface in the last 100 ns simulations of M2 (orange line) and M10 (sky blue line).

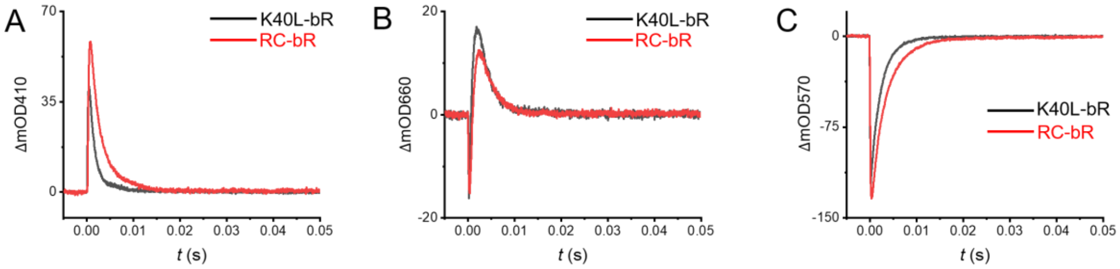

Figure S17. Light-induced transient absorption changes by K40L mutant in the native membranes in the M **(A)** and O state **(B)** and the recovery trajectory towards the bR ground state **(C)** at 410 nm, 660 nm, and 570 nm, respectively.

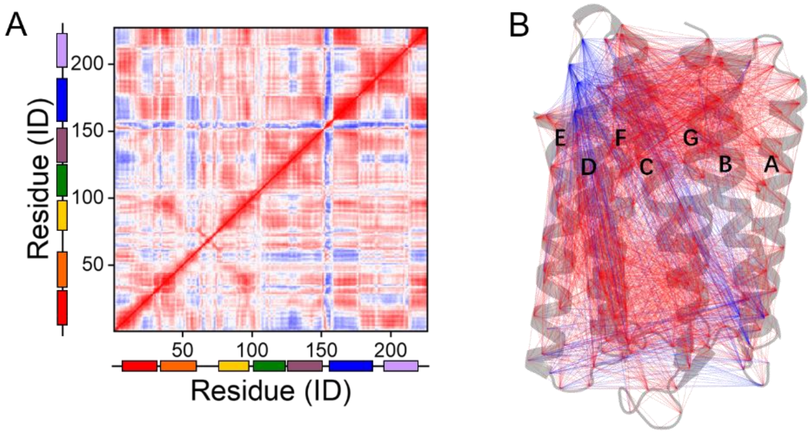

Figure S18. (A) Dynamic cross-correlation map and **(B)** dynamic cross-correlation network analysis of residue–residue cross-correlations of the Cα atoms for M9 (Membrane containing the K40L-bR trimer, POPC, S-TGA-1, and PGP-Me lipids).

Table S1. Summary of the MD simulations performed.

Sim	Simulated Model	System	Duration (ns)
Sim1	bR-S-TGA-1_PGP-Me (M1)	bR trimer + TGA[2] + DPG[3] + POPC bilayer[1]	1000 × 2
Sim2	bR-POPC (M2)	bR trimer + POPC bilayer[1]	1000 × 2
Sim3	bR-S-TGA-1 (M3)	bR trimer + TGA[2] + POPC bilayer[1]	1000 × 2
Sim4	bR-PGP-Me (M4)	bR trimer + DPG[3] + POPC bilayer[1]	1000 × 2
Sim5	bR-W80A (M5)	W80A-bR trimer + TGA[2] + DPG[3] + POPC bilayer[1]	1000 × 2
Sim6	bR-K129L (M6)	K129L-bR trimer + TGA[2] + DPG[3] + POPC bilayer[1]	1000 × 2
Sim7	bR-POPC-W80A (M7)	W80A-bR trimer + POPC bilayer[1]	1000 × 2
Sim8	bR-POPC-K129L (M8)	K129L-bR trimer + POPC bilayer[1]	1000 × 2
Sim9	bR-K40L (M9)	K40L-bR trimer + TGA[2] + DPG[3] + POPC bilayer[1]	1000 × 2
Sim10	bR-POPC-K40L (M10)	K40L-bR trimer + POPC bilayer[1]	1000 × 2

[1] These simulations used a preformed lipid bilayer.
[2] TGA represents the S-TGA-1.
[3] DPG represents the PGP-Me.

Table S2. The root mean square fluctuations (RMSF) for the individual helices during M1 to M4 whole simulations.

	RMSF (Å)			
**Helix**	**M1**	**M2**	**M3**	**M4**
**A**	0.57	0.79	0.60	0.66
**B**	0.50	0.67	0.52	0.52
**C**	0.52	0.69	0.58	0.57
**D**	0.44	0.78	0.48	0.64
**E**	0.50	0.85	0.67	0.63
**F**	0.62	0.78	0.74	0.67
**G**	0.59	0.67	0.63	0.64

Table S3. Distance between the key residues and retinal in the last 100 ns simulations of M1 (Membrane containing the bR trimer, POPC, S-TGA-1, and PGP-Me lipids) and M2 (Membrane containing the bR trimer and POPC lipids only).

Distance/Å	Y83-E194	R82-A51	E194-E204	R134-E194
M1	2.68±0.18	13.08±0.37	2.64±0.15	2.92±0.36
M2	2.71±0.31	13.31±0.36	5.00±0.44	2.87±0.13
M1 (second simulation)	2.70±0.23	13.49±0.35	2.62±0.10	2.95±0.33
M2 (second simulation)	2.69±0.15	13.55±0.52	4.82±0.29	2.89±0.26

Table S4. Distance between the key residues and retinal in the last 100 ns simulations of M5 (Membrane containing the W80A-bR trimer, POPC, S-TGA-1, and PGP-Me lipids) and M7 (Membrane containing the W80A-bR trimer and POPC lipids only).

Distance/Å	Y83-E194	R82-A51	E194-E204
M5	2.90±0.62	13.20±0.47	5.19±0.73
M7	4.44±0.76	12.09±0.37	3.15±0.88
M5 (second simulation)	2.88±0.61	13.49±0.35	5.05±0.61
M7 (second simulation)	4.12±0.85	11.92±0.35	2.92±0.68

Table S5. Distance between the key residues and retinal in the last 100 ns simulations of M6 (Membrane containing the K129L bR trimer, POPC, S-TGA-1, and PGP-Me lipids) and M8 (Membrane containing the K129L-bR trimer and POPC lipids only).

Distance/Å	R134-E194	E194-E204
M6	3.60±0.96	3.41±0.93
M8	2.97±0.42	5.03±0.79
M6 (second simulation)	3.27±0.55	3.44±0.89
M8 (second simulation)	2.83±0.12	4.87±0.54

Table S6. The Cα root mean square deviation (RMSD) for the cytoplasmic ends of helices A, B, D, and E during M1, M2, M9 (Membrane containing the K40L-bR trimer, POPC, S-TGA-1, and PGP-Me lipids), and M10 (Membrane containing the K40L-bR trimer and POPC lipids only), regarding a late M-state crystal structure (PDB ID: 1FBB). The smaller RMSDs indicate larger openings.

Helix	RMSD (Å)			
	M1	M2	M9	M10
A	2.42±0.18	2.72±0.09	2.66±0.18	2.72±0.08
B	1.13±0.19	2.23±0.09	1.07±0.14	2.15±0.08
D	1.67±0.05	1.81±0.06	1.71±0.07	1.80±0.06
E	1.52±0.13	2.08±0.12	1.51±0.12	2.10±0.13

Table S7. The forward and reverse primers of *K40L-bop* and *K129L-bop*.

primers		Sequences (5′-3′)
forward	P1	CGGGATCCGACGTGAAGATGGGG
reverse	P2	GCCAAGCTTCTAGATCAGTCGCTG
K40L	P3	GGCGTAGAATTTCAGTGCATCTGGG
	P4	CCCAGATGCACTGAAATTCTACGCC
K129L	P5	GGCGCACTGACGCTGGTCTACTCGTAC
	P6	GTACGAGTAGACCAGCGTCAGTGCGCC

References

1. Cao, Z.; Ding, X.; Peng, B.; Zhao, Y.; Ding, J.; Watts, A.; Zhao, X. Novel expression and characterization of a light-driven proton pump archaerhodopsin 4 in a Halobacterium salinarum strain. *Biochim. Biophys. Acta*. **2015**, 1847, 390-398.
2. Krebs, M.P.; Mollaaghababa, R.; Khorana, H.G. Gene replacement in Halobacterium halobium and expression of bacteriorhodopsin mutants. *Proc. Natl. Acad. Sci. U.S.A.* **1993**, 90, 1987-1991.
3. Krebs, M.P.; Hauss, T.; Heyn, M.P.; RajBhandary, U.L.; Khorana, H.G. Expression of the bacterioopsin gene in Halobacterium halobium using a multicopy plasmid. *Proc. Natl. Acad. Sci. U.S.A.* **1991**, 88, 859-863.
4. Cline, S.W.; Lam, W.L.; Charlebois, R.L.; Schalkwyk, L.C.; Doolittle, W.F. Transformation methods for halophilic archaebacteria. *Can. J. Microbiol.* **1989**, 35, 148-152.
5. Oesterhelt, D.; Stoeckenius, W. Rhodopsin-like Protein from the Purple Membrane of Halobacterium halobium. *Nat. New Biol.* **1971**, 233, 149-152.
6. Ming, M.; Lu, M.; Balashov, S.P.; Ebrey, T.G.; Li, Q.; Ding, J. pH dependence of light-driven proton pumping by an archaerhodopsin from Tibet: comparison with bacteriorhodopsin. *Biophys. J.* **2006**, 90, 3322-3332.
7. Luecke, H.; Schobert, B.; Richter, H.T.; Cartailler, J.P.; Lanyi, J.K. Structural Changes in Bacteriorhodopsin During Ion Transport at 2 Angstrom Resolution. *Science* **1999**, 286, 255-260.
8. Luecke, H.; Schobert, B.; Cartailler, J.-P.; Richter, H.-T.; Rosengarth, A.; Needleman, R.; Lanyi, J.K. Coupling photoisomerization of retinal to directional transport in bacteriorhodopsin. *J. Mol. Biol.* **2000**, 300, 1237-1255.
9. Lanyi, J.K.; Schobert, B. Crystallographic Structure of the Retinal and the Protein after Deprotonation of the Schiff Base: The Switch in the Bacteriorhodopsin Photocycle. *J. Mol. Biol.* **2002**, 321, 727-737.
10. Lanyi, J.K. Bacteriorhodopsin. *Annu. Rev. Physiol.* **2004**, 66, 665-688.
11. Wang, T.; Sessions, A.O.; Lunde, C.S.; Rouhani, S.; Glaeser, R.M.; Duan, Y.; Facciotti, M.T. Deprotonation of D96 in Bacteriorhodopsin Opens the Proton Uptake Pathway. *Structure* **2013**, 21, 290-297.
12. Wickstrand, C.; Nogly, P.; Nango, E.; Iwata, S.; Standfuss, J.; Neutze, R. Bacteriorhodopsin: Structural Insights Revealed Using X-Ray Lasers and Synchrotron Radiation. *Annu. Rev. Biochem.* **2019**, 88, 59-83.
13. Schobert, B.; Brown, L.S.; Lanyi, J.K. Crystallographic Structures of the M and N Intermediates of Bacteriorhodopsin: Assembly of a Hydrogen-bonded Chain of Water Molecules Between Asp-96 and the Retinal Schiff Base. *J. Mol. Biol.* **2003**, 330, 553-570.
14. Nango, E.; Royant, A.; Kubo, M.; Nakane, T.; Wickstrand, C.; Kimura, T.; Tanaka, T.; Tono, K.; Song, C.; Tanaka, R.; et al. A three-dimensional movie of structural changes in bacteriorhodopsin. *Science* **2016**, 354, 1552-1557.
15. Neutze, R.; Pebay-Peyroula, E.; Edman, K.; Royant, A.; Navarro, J.; Landau, E.M. Bacteriorhodopsin: a high-resolution structural view of vectorial proton transport. *Biochim. Biophys. Acta*. **2002**, 1565, 144-167.
16. Wickstrand, C.; Dods, R.; Royant, A.; Neutze, R. Bacteriorhodopsin: Would the real structural intermediates please stand up? *Biochim. Biophys. Acta*. **2015**, 1850, 536-553.
17. Lanyi, J.K.; Schobert, B. Mechanism of Proton Transport in Bacteriorhodopsin from Crystallographic Structures of the K, L, M1, M2, and M2' Intermediates of the Photocycle. *J. Mol.*

Biol. **2003**, 328, 439-450.
18. Lanyi, J.K. Mechanism of proton transport from crystallographic structures of the nine states of the bacteriorhodopsin photocycle. *Biochim. Biophys. Acta*. **2004**, 1658, 78-78.
19. Lanyi, J.K. Proton transfers in the bacteriorhodopsin photocycle. *Biochim. Biophys. Acta*. **2006**, 1757, 1012-1018.
